# Closing the loop in a constructed wetland for the improvement of metal removal: the use of *Phragmites australis* biomass harvested from the system as biosorbent

**DOI:** 10.1007/s11356-020-11291-0

**Published:** 2020-10-29

**Authors:** Elisabetta Bianchi, Andrea Coppi, Simone Nucci, Alexandra Antal, Chiara Berardi, Ester Coppini, Donatella Fibbi, Massimo Del Bubba, Cristina Gonnelli, Ilaria Colzi

**Affiliations:** 1 Department of Biology, Università degli Studi di Firenze, via Micheli 1, 50121 Florence, Italy; 2 GIDA S.p.A., via di Baciacavallo, 36, 59100 Prato, Italy; 3 Department of Chemistry, Università degli Studi di Firenze, Via della Lastruccia 3, 50019 Sesto Fiorentino, Florence Italy

**Keywords:** Biosorption, Circular economy, Columns, Trace metal removal, Plant dead biomass

## Abstract

Among the numerous clean-up techniques for water treatment, sorption methods are widely used for the removal of trace metals. *Phragmites australis* is a macrophyte commonly used in constructed wetlands for water purification, and in the last decades, its use as biosorbent has attracted increasing attention. In view of a circularly economy approach, this study investigated improvement of trace metal removal by recycling the biomass of *P. australis* colonizing a constructed wetland, which operates as post-treatment of effluent wastewater from an activated sludge plant serving the textile industrial district of Prato (Italy). After the annual mowing of the reed plants, the biomass was dried and blended to derive a sustainable and eco-friendly biosorbent and its sorption capacity for Fe, Cu, and Zn was investigated comparing the batch system with the easier-to-handle column technique. The possibility of regeneration and reuse of the biosorbent was also evaluated. The biomaterial showed an interesting sorption capacity for Cu, Fe, and Zn, both in batch and in column experiments, especially for Fe ions. The immobilization of the biosorbent in column filters induced some improvement in the removal efficiency, and, in addition, this operation mode has the advantage of being much more suitable for practical applications than the batch process.

## Introduction

In this century, water pollution is of increasing concern to society, in addition exacerbated by the problem of water availability as a result of climate changes. Therefore, preventing the sources of water pollution further reaching the water bodies and cleaning the already polluted wastewaters are equally acquiring compulsory importance (Newete and Byrne 2016).

Trace metals represent the most common pollutants of high concern, since they cannot be biodegraded and can therefore accumulate in waters and soils (Ali et al. 2013). Into aquatic bodies, metals are mainly released from anthropogenic sources, even though in particular environments, their natural origin can be significant (Schwarzenbach et al. 2006). Excessive levels of such elements can cause serious toxic effects at any level of the ecosystem, including several health problems (Ali et al. 2013; Rahman and Singh 2019).

Conventional physical and/or chemical methods for the treatment of wastewater are expensive and not eco-friendly (Sharma et al. 2014); therefore, in the last decades, several methodologies have been developed for cleaning water. Among them, constructed wetlands (CWs) are effective systems based on the use of macrophytes that exploit their high growth rate and large root apparatus for the direct uptake of pollutants (Fibbi et al. 2012; Mitsch 2012; Newete and Byrne 2016) and, above all, for the synergistic interactions with the microbial communities of CWs (Sacco et al. 2006; Truu et al. 2009). Such simple and low-cost technology is extensively used for the treatment of domestic sewage, as well as of industrial and agricultural wastewater (Gorgoglione and Torretta 2018; Masi et al. 2018). In fact, CWs can efficiently lower a wide range of pollutants, including metals that are removed thanks to precipitation and complexation mechanisms (Marchand et al. 2010), and to the considerable ability of macrophytes to accumulate such elements from water (Ali et al. 2013; Sharma et al. 2014).

Recently, another economic and eco-friendly alternative technology that is emerging for wastewater treatment is represented by the use of adsorbents derived from different biological materials, preferably low-cost wastes (Fu and Wang 2011; Srivastava et al. 2015; Del Bubba et al. 2020). A wide range of natural biomasses with sorptive properties has been tested for metal removal from contaminated water (e.g., Gupta et al. 2015). Among them, plant-derived materials are one of the favorite substrates, since they are renewable, abundant, easy to handle, cost-effective, and do not present many problems for their final disposal (Rai 2009; Ahluwalia and Goyal 2007; Srivastava et al. 2015). Biosorbents obtained from dried plant biomass, including those derived from agricultural waste products (Bhabhatnagar and Sillanpaa 2010; Castro et al. 2017; Petrovic et al. 2017), are the most frequently proposed for the removal of trace metals from wastewater (Guyo and Moyo 2017; Kamar et al. 2017; Saha et al. 2017; Sao et al. 2017, Colzi et al. 2018). Valid candidates, as non-living biomass sources, have been identified even in several macrophyte species (Bunluesin et al. 2007; Halaimi et al. 2014; Módenes et al. 2017; Rodrigues et al. 2017; Yi et al. 2017), and among them, the dried biomass of *Phragmites australis* (Cav.) Trin. ex Steudel. is attracting increasing attention. Actually, *P. australis*, a perennial helophyte grass generally found in tropical and temperate wetlands (Batty 2003), is the plant species most commonly employed in CWs for the wastewater treatment, able to enhance the removal of contaminants by adsorption, accumulation, and oxidation (Lee and Scholz 2007). Besides the active role of *P. australis* in CWs, also its non-living biomass has been reported to have a remarkable sorption capacity, coupled to easy desorption and regeneration by acid elution, for Cu, Cd, Ni, Pb, and Zn, thanks to its high concentrations of lignin and cellulose (Southichak et al. 2006). Further batch experiments showed that also Hg was effectively bioremoved by *P. australis* dried biomass (Kankiliç et al. 2018), together with Cr, by the powder derived from *P. australis* roots (Akunwa et al. 2014). Moreover, considering the worldwide distribution of *P. australis* in nature and its use in CWs, where the management of mature plants is required, these appropriate features make *P. australis* dried biomass a good candidate as renewable biosorbent material.

In this study, we devised a strategy to exploit the biomass of *P. australis* harvested in a CW working as tertiary treatment of a membrane bioreactor wastewater treatment plant (WWTP) designed to pre-treat a mixture of landfill leachates prior to being conveyed in an activated sludge WWTP (Coppini et al. 2019). Although CW is able to lower Cu, Fe, and Zn concentrations (Coppini et al. 2019), they still remain above the limits established by the Italian law for discharge into surface waters (Legislative Decree 152/06). Despite CW effluent is then further treated in the activate sludge WWTP and metals are removed, the use of harvested CW biomass is here proposed as an alternative way to optimize the CW treatment. More precisely, this study aims at exploiting the dried biomass of *P. australis* plants, coming from the annual mowing of the CW, to derive a sustainable and eco-friendly biosorbent for the removal of the abovementioned elements from the CW effluent. The approach is in accordance with a circular economy model, since the biomaterial comes from the CW, thus giving it a second life before its disposal as waste. The biosorbent derived from the *P. australis* dried biomass was tested for Cu, Fe, and Zn removal both in batch mode and in an easier-to-handle column system. Some papers in literature report for *P. australis* biomass successful trials in batch experiments (see references above); however, to the best of our knowledge, only one previous study used a column system to investigate the sorption ability of *P. australis* dried biomass for Cr(VI) removal (Lagiopoulos et al. 2017). Therefore, this is the first report that investigates the possible use of *P. australis* dried biomass recycled from a CW for the generation of a column system to optimize the removal of Cu, Fe, and Zn. The results will be of fundamental importance to suggest an easy, effective, and “green” strategy that any CW-based treatment plant could experience for the cleaning of the most precious resource of the earth, water.

## Materials and methods

### Biomass and synthetic wastewater preparation

*Phragmites australis* plants were collected from the CW at the Calice WWTP (Prato, Italy) for urban and industrial wastewater treatment, managed by the company Gestione Impianti Depurazione Acque (GIDA S.p.A.). In the lab, plant shoots were cut, cleaned with distillated water, and dried at 60 °C in an oven. Once dried, the plant biomass was ground by an electric grinder and sieved to 0.5 mm to obtain a homogeneous powder.

For the experiment, a synthetic wastewater (SWW) with pH and metal concentration similar to the real influent of the Calice WWTP (data provided by GIDA S.p.A.) was prepared. The necessary amount of metal salts (Cu(NO_3_)_2_·5H_2_O, FeSO_4_·7H_2_O, and Zn(NO_3_)_2_·6H_2_O) was dissolved in deionized water to obtain the corresponding metal ion concentration in the synthetic water: Cu(II) 0.3 mg L^−1^, Fe(II) 8 mg L^−1^, and Zn(II) 2 mg L^−1^. The pH of the solution was adjusted with NH_4_OH to simulate the value of the real wastewater influent of the Calice WWTP (7.8 ± 0.2).

### Batch experiment

Batch experiments were performed using 250-mL conical flasks (9 replicates) containing 100 mL of SWW and about 8.7 g of *P. australis* dried biomass. The biosorbent was allowed to come into contact with the solution for 20 min on a magnetic stirrer. Subsequently, the solution was filtered through 0.45-μm filter paper, and the metal concentrations were determined as described below, both in the supernatant and in the biosorbent.

### Column filtration experiment

**Fig. 1:**
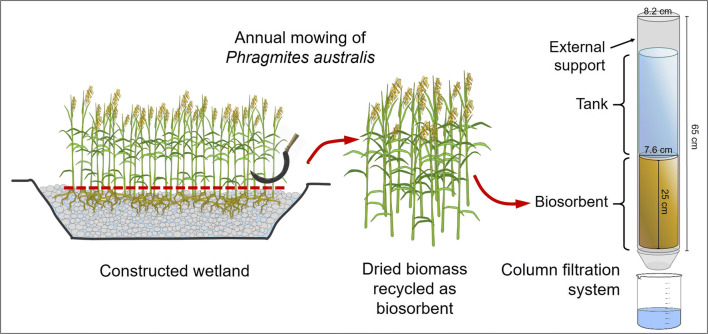
Experimental setup for column filtration studies

The column filtration system was realized at the lab scale (Fig. 1), and was composed of a plexiglass cylinder (height 25 cm, internal diameter 7.6 cm) completely filled with dried *P. australis* biomass (about 175 g for each column). Perforated caps with disks of filter paper (0.45 μm) were placed at the top and at the bottom of the column, to enable a uniform distribution of the inlet and outlet flow of wastewater and to support the biosorbent, preventing any loss. For convenience, the column was inserted into a support cylinder with a larger diameter (Plexiglas, height 65 cm, internal diameter 8.2 cm). The column was equipped with a graduated Plexiglas reservoir, which was placed above the top filter. The column experiments were performed by adding 2 L of SWW to the tank and letting it percolate through the *P. australis* biosorbent filter. After the filtration, about 2 g of fresh weight (f.w.) *P. australis* biomass (corresponding to 0.5 g of dry weight (d.w.)) and SWW (90 mL) aliquots were collected and used for the determination of metal concentrations. The filtration through the biosorbent was repeated 4 times (F1–F4), adding 2 L of new SWW every time, for a total of 8 L of filtered liquid through each column. Prior to each filtration with the SWW, the columns were washed with 2 L of deionized water in order to check the absence of element release from the plant biomass to the liquid. Moreover, aliquots of plant biomass and SWW before the beginning of the filtration experiments were sampled and used for the determination of metal concentration at the beginning of the experiment (*t*_0_).

The experiments were conducted at room temperature (25 ± 2 °C), and the percolation rate was 100 mL per min.

### Desorption and reuse of the biosorbent

A desorption experiment was conducted by passing 1 L of 0.1 M HCl (regeneration solution) directly through the columns containing the *P. australis* biosorbent. Afterwards, 2 L of deionized water was let to percolate through the column to remove any trace of acid. Aliquots of the regeneration solution (90 mL) and biosorbent (0.5 g d.w.) were collected after each leaching and used for the determination of metal concentrations. The whole desorption procedure was repeated three times (D1–D3), adding 1 L of new regeneration solution every time, for a total of 3 L of diluted HCl used for each column system.

At the end of the desorption procedure, the reusability of the biosorbent was tested by carrying out a new percolation experiment. Two consecutive filtrations, using new SWW every time, were performed, following the same procedure described in “Desorption and reuse of the biosorbent” section.

### Determination of metal concentrations

The concentrations of Cu, Fe, and Zn in *P. australis* biosorbent and liquid samples (SWW and regeneration solution) were determined through mineralization in a microwave digestion system (Mars 6, CEM) and subsequent analysis by atomic absorption spectrometry (PinAAcle 500, Perkin Elmer). In particular, the plant biomass was blotted dry on paper to remove any residue of SWW and then dried in the oven at 60 °C for at least 24 h. About 0.1 g d.w. of material was digested using 10 mL of HNO_3_ 69% (according to Bettarini et al. 2019), and at the end of mineralization, the volume was adjusted to 25 mL with Milli-Q water (Millipore, Burlington, MA, USA).

All the liquid samples were filtered through a 0.45-μm filter paper, in order to separate possible suspended material that could influence the metal measurements. Aliquots of 20 mL were used for the digestion by adding 1.5 mL of HNO_3_ 69% and 1 mL of HCl 37% (according to US EPA3015a), and the final volume was adjusted to 25 mL with Milli-Q water.

The percentage of metal removal by *P. australis* biomass was calculated based on the decrease of element concentrations in the SWW (*R*%_SWW_) after batch/column experiments, according to Eq. (1):

1 $$ \mathrm{R}{\%}_{\mathrm{SWW}}=\frac{{\mathrm{C}}_{\mathrm{SWW}}^{{\mathrm{t}}_0}-{\mathrm{C}}_{\mathrm{SWW}}^{{\mathrm{t}}_{\mathrm{end}}}}{{\mathrm{C}}_{\mathrm{SWW}}^{{\mathrm{t}}_0}}\times 100 $$

where

$$ {C}_{\mathrm{SWW}}^{t_0} $$
metal concentration (mg L^−1^) in the SWW at *t*_0_ (before batch/column experiments)
$$ {C}_{\mathrm{SWW}}^{{\mathrm{t}}_{\mathrm{end}}} $$
metal concentration (mg L^−1^) in the SWW after batch experiment and each one of the percolation/desorption cycles.

Moreover, the removal percentage of metals was calculated as reported in Colzi et al. (2018), based on the concentration found in the *P. australis* biomass (*R*%_biomass_) after batch/column experiment, according to Eq. (2):

2 $$ \mathrm{R}{\%}_{\mathrm{biomass}}=\frac{{\mathrm{M}}_{\mathrm{biomass}}^{{\mathrm{t}}_{\mathrm{n}+1}}-{\mathrm{M}}_{\mathrm{biomass}}^{{\mathrm{t}}_{\mathrm{n}}}}{{\mathrm{C}}_{\mathrm{SWW}}^{{\mathrm{t}}_0}\times \mathrm{V}}\times 100 $$

where

$$ {M}_{\mathrm{biomass}}^{t_n} $$
metal amount (μg) in the biosorbent before (i) the batch experiment or (ii) each one of the percolation/desorption cycles.
$$ {M}_{\mathrm{biomass}}^{t_{n+1}} $$
metal amount (μg) in the biosorbent after (i) the batch experiment or (ii) each one of the column percolation/desorption cycles.
$$ {C}_{\mathrm{SWW}}^{t_0} $$
metal concentration (μg L^−1^) in the SWW at *t*_0_ (before batch/column experiments)
*V*: volume of the SWW (L) used for the batch experiment and each column percolation/desorption cycles.

### Statistics

Plot drawing and statistical analysis were conducted using GraphPad Prism 8 for Windows. One-way ANOVA was performed to compare mean values, and Tukey post hoc test was used for a posteriori comparison of individual means (with at least *p* < 0.05 as significance level).

## Results and discussion

### Batch experiment

The batch experiment was performed allowing the *P. australis* biomass to come into contact with the SWW for 20 min. Since a rapid sorption is important for improving the process efficiency in practical application, we purposely decided to test the biosorption capacity of the *P. australis* biosorbent only at a short contact time. Rapid metal biosorption in batch tests has been reported for several kinds of biomass, reaching the kinetic equilibrium within 20 min (e.g., Keskinkan et al. 2004; Bunluesin et al. 2007; Kariuki et al. 2017; Abdić et al. 2018).

Metal concentrations (Cu, Fe, and Zn) in SWW and *P. australis* biomass determined before and after the batch sorption are reported in Table 1. In the synthetic solution, all metal concentrations significantly decreased after the batch experiment, and corresponding concentration increases were observed in the *P. australis* biomass. The plant biomass showed an efficient sorption capacity, reaching the highest percentage of removal determined in the SWW (*R*%_SWW_, Eq. (1)) for Fe (about 95%), followed by Zn (about 73%) and Cu (about 61%). The removals calculated on the basis of the concentrations determined in the plant biomass (*R*%_biomass_, Eq. (2)) were in agreement with *R*%_SWW_ data and confirmed the same trend. A similar metal biosorption capacity of *P. australis* biomass was already observed in the case of Hg, reaching about 75% of removal within 20 min (Kankiliç et al. 2018). Rapid and efficient metal biosorption has been reported also for other macrophyte-based biomaterials. For instance, Bunluesin et al. (2007) reported an 80% biosorption efficiency of *Hydrilla verticillata* biomass during the first 3–5 min of batch sorption for Cd removal, reaching the equilibrium within 20 min of contact time, similarly to the case of Zn, Pb, and Cu sorption by *Ceratophyllum demersum* (Keskinkan et al. 2004), and Cu, Pb, and Cr by *Sagittaria trifolia* (Zhang et al. 2018). Residual Fe concentration in the SWW (0.6 mg L^−1^) was much below the limits for wastewater discharge in surface waters (2 mg L^−1^) indicated by Italian law (Legislative Decree 152/06), whereas Cu and Zn SWW concentrations after the batch sorption experiment (0.11 and 0.53 mg L^−1^) were around the corresponding threshold values (0.1 mg L^−1^ for Cu and 0.5 mg L^−1^ for Zn).

**Table 1:** Metal concentration (mean of 9 replicates ± SD) in the synthetic wastewater (SWW, mg L^−1^) and in the *P. australis* biomass (μg g^−1^ d.w.) and percentages of metal removal (%) after the batch sorption for 20 min

Metals									
	Cu			Fe			Zn		
SWW (mg L^−1^)									
Before sorption	0.30	±	0.01a	8.1	±	0.5a	2.12	±	0.08a
After sorption	0.11	±	0.01b	0.6	±	0.1b	0.53	±	0.03b
Biomass (μg g^−1^ d.w.)									
Before sorption	2.2	±	0.3b	39	±	5b	32	±	4b
After sorption	4.3	±	0.3a	126	±	4a	49	±	2a
Removal (%)									
*R*%_SWW_	61	±3 c	3 c	93	±	1 a	73	±	1 b
*R*%_biomass_	60	±5 c	5 c	95	±	4 a	73	±	4 b

Significant differences before and after the sorption appear with different letters (at least *p* < 0.05)

### Column filtration experiment

Since the dead biomass of *P. australis* showed a remarkable sorption capacity for the studied elements in short-term batch experiment, in view of a greater optimization of metal removal for practical application, a column filtration system was tested. Usually, thinking about an upgrading of the process at a larger scale, column systems are considered preferable for a continuous wastewater treatment. Moreover, they allow a more efficient and economical utilization of the biosorbents than a batch mode, avoiding all the necessary operations for separating the biomass from the liquid after treatment (Vinodhini and Das 2010). In addition, our column system was conceived for a smart handling, since the removable filtration cylinder containing the biosorbent dovetails with the external support column and can be easily extracted and replaced when saturated.

**Fig. 2:**
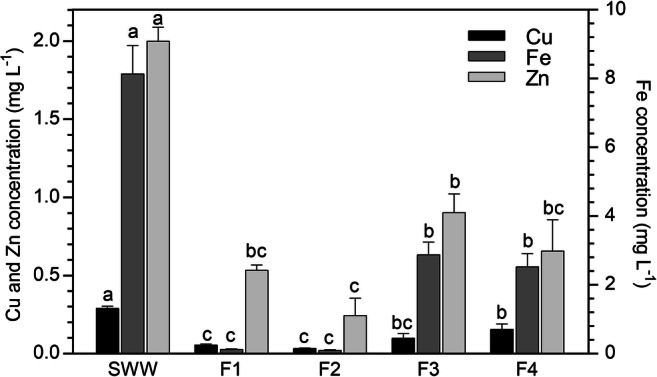
Metal concentration (mg L^−1^) in the synthetic water before (SWW) and after the percolation (F1–F4) through the column system (mean of 15 replicates ± SE). Significant differences among the filtrations appear with different letters (at least *p* < 0.05)

As illustrated in Fig. 2, the filtration of SWW through the biosorbent resulted in a statistically significant decrease in the concentrations of all metals in the column effluent, compared with the influent solution. Such significant reduction of the metal concentrations was observed for after all the 4 performed filtrations. Therefore, the column system showed a high efficiency in reducing the metal concentrations from the SWW, reaching the best performances in F1 and F2, these latter percolation cycles being the ones that provided the lowest metal concentrations in the column outlet. An increasing trend of the column effluent concentrations was then observed (i.e., in F3 and F4), evidencing a tendency to saturation of the biosorbent. With increasing the volume of treated SWW the sorption efficiency was lower, however the column system could be used with a pre-treatment purpose, followed by a subsequent additional depuration through the CW plant.

**Fig. 3:**
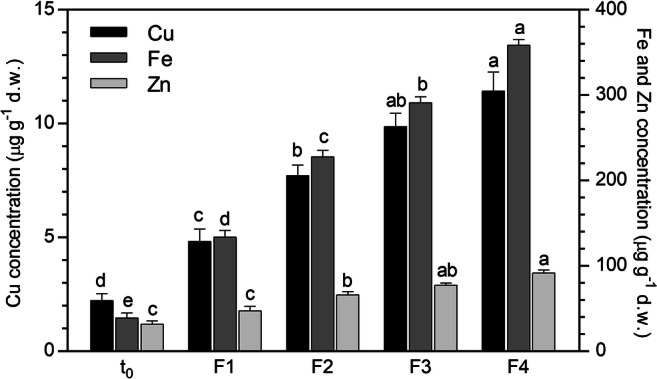
Metal concentration (μg g^−1^) in the *P. australis* biomass before (*t*_0_) and after the percolation (F1–F4) of SWW through the column system (mean of 15 replicates ± SE). Significant differences among the filtrations appear with different letters (at least *p* < 0.05)

In the *P. australis* biomass (Fig. 3), the concentration of all the metals increased with increasing the number of consecutive filtrations, even though the amount of elements retained by the column after each filtration tended to decrease from F1 to F4. Obviously, the initial higher sorption capacity is due to the abundance of free binding sites, which decrease during the filtration process.

The removals calculated on the basis of the concentrations determined in the SWW (*R*%_SWW_, Eq. (1)) or in the biomass (*R*%_biomass_, Eq. (2)) were found to be in agreement with each other. These values were in the ranges of about 46–80%, 70–100%, and 65–85% for Cu, Fe, and Zn, respectively (Table 2). Considering that for the first percolation cycle of the column experiment the ratio between the biomass weight and the SWW volume was the same of the batch study (i.e., 87 g L^−1^), it is possible to compare the two treatment approaches, evidencing removal percentages similar (for Fe and Zn) or higher (for Cu) than those obtained in the batch study.

**Table 2:** Percentages of metal removal of *P. australis* biomass (mean of 15 replicates ± SD) after each filtration through the column system

Metals									
	Cu			Fe			Zn		
*R*%_SWW_									
F1	81	±	2 aB	98	±	2 aA	72	±	2 bC
F2	89	±	1 bB	99	±	2 aA	87	±	5 aB
F3	66	±	9 cdA	64	±	4 bA	52	±	6 cA
F4	46	±	5 dB	69	±	4 bA	65	±	9 bcAB
*R*%_biomass_									
F1	79	±	6 aB	102	±	8 aA	72	±	2 aB
F2	86	±	5 aB	101	±	4 aA	86	±	8 aB
F3	65	±	7 bB	68	±	3 aA	52	±	4 aB
F4	46	±	9 cB	72	±	2 aA	65	±	4 aB

Significant differences appear with different letters, small for the comparison among the filtrations and capital for the comparison among the metals (at least *p* < 0.05)

As for the comparison with previously published biosorption data, most of the biomaterials reported in literature for metal removal with column systems are based on agricultural wastes recycled from the food production chain (e.g., Pérez Marín et al. 2009; Iqbal et al. 2013; Chao et al. 2014; Abdolali et al. 2017; Dong and Lin 2017). A fewer number of studies took into consideration plant biomass (Karunasagar et al. 2005; Stanovych et al. 2019), and, to the best of our knowledge, only one paper tested *P. australis* dried biomass in column mode for the removal of Cr(VI) (Lagiopoulos et al. 2017). The comparison of the results obtained here with those previously reported in the literature is complicated by the fact that the metal removal efficiencies, in addition to the type of biosorbent (often chemically modified prior its use as biosorbent), are strongly affected by a number of operating parameters such as the percolation rate and the metal concentration of the influent solution (Abdolali et al. 2017). In this study, the concentrations of metals accumulated in the biomaterial resulted quite lower than the corresponding values determined elsewhere (Vijayaraghavan et al. 2005; Muhamad et al. 2010; Hasfalina et al. 2012; Acheampong et al. 2013; Chao et al. 2014; Martín-Lara et al. 2016; Talebian et al. 2016; Abdolali et al. 2017; Dong and Lin 2017; Tariq et al. 2019). In particular, the highest values reported in the literature were obtained with low percolation rates (a few mL min^−1^) and high metal concentrations in the influent solution (from tens to hundreds of mg L^−1^). However, it is evident that these experimental conditions are very far from those that should be achieved in a WWTP, where the concentrations of metal to be treated are normally lower and the water flow rates on the other hand much higher. Moreover, in our tests, the columns were not saturated, whereas literature data commonly refer to maximum sorptions obtained under breakthrough conditions. Furthermore, the biomaterial used did not received a chemical pretreatment to improve its sorption potential in order to limit costs and the impact on the environment. It should also be noted that in some breakthrough studies performed in column packed with biosorbents, metal sorption is surprisingly reported to be far greater than the quantities of metal applied to the column itself (e.g., Acheampong et al. 2013; Abdolali et al. 2017).

### Desorption and reuse of the biosorbent

The regeneration of the metal-loaded *P. australis* biosorbent and the possibility to reuse the column system prior to discharge the biomass is a very important aspect in view of a more sustainable and cost-effectiveness process. Moreover, the possible non-destructive recovery of the metal by desorbing agents would give to the process an added economic value (Vijayaraghavan and Yun 2008).

**Fig. 4:**
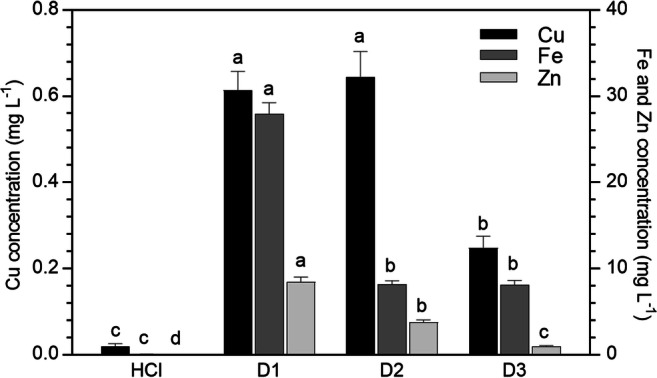
Metal concentration (mg L^−1^) in the HCl solution before (HCl) and after the percolation (D1–D3) through the column system (mean of 15 replicates ± SE). Significant differences among the desorption cycles appear with different letters (at least *p* < 0.05)

The column regeneration was performed with diluted HCl solution (regeneration solution), and the metal concentration was determined after the percolation through the *P. australis* biosorbent (Fig. 4). In general, a gradual decrease in the metal concentrations was observed with increasing the number of desorption leaching. The highest values were found after the first desorption (D1). After the second percolation with new regeneration solution (D2), the Cu concentration in the eluate was the same than after D1, whereas Fe and Zn concentrations were significantly lower. Finally, in the regeneration solution used for the third desorption (D3), the lowest metal concentrations were found, with the exception of Fe, which did not show any significant decrease in comparison with D2.

**Fig. 5:**
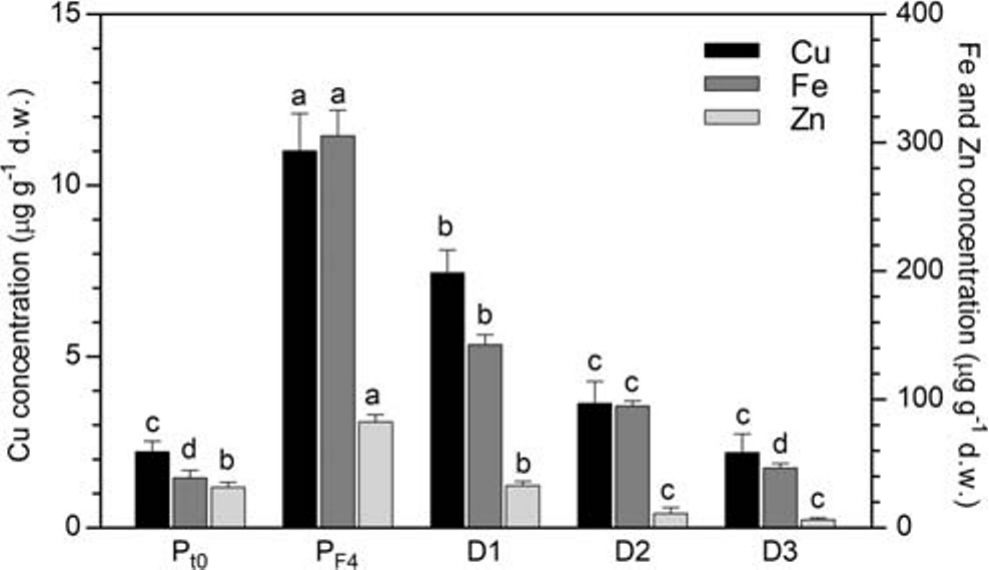
Metal concentration (μg g^−1^) in the *P. australis* biosorbent after the desorption (D1–D3) with HCl (mean of 15 replicates ± SE). The metal concentration in the *P. australis* biomass before (*P*_*t*0_) and after 4 consecutive filtrations of SWW (*P*_F4_) is also reported in the graph. Significant differences among *P*_*t*0_, *P*_F4_, and the desorption filtrations appear with different letters (at least *p* < 0.05)

In Fig. 5, the metal concentrations in *P. australis* biosorbent after three desorption filtrations with the regeneration solution are reported. Moreover, the figure shows the initial metal concentration in the *P. australis* biomass (*P*_*t*0_) and the saturated level, reached after 4 consecutive filtrations of SWW (*P*_F4_). Starting from the metal saturated biomass (*P*_F4_), a progressive significant decrease of the metal concentrations in the biosorbent was found with the increase in the number of desorption filtrations. In the case of Fe, the biomass resulted completely desorbed after three filtrations with the regeneration solution, since the concentrations determined in the biosorbent were not significantly different from the initial values within the *P. australis* biomass (*P*_*t*0_). As for Cu and Zn, the same result was observed already after two (D2) and one desorption with the regeneration solution (D1), respectively.

**Fig. 6:**
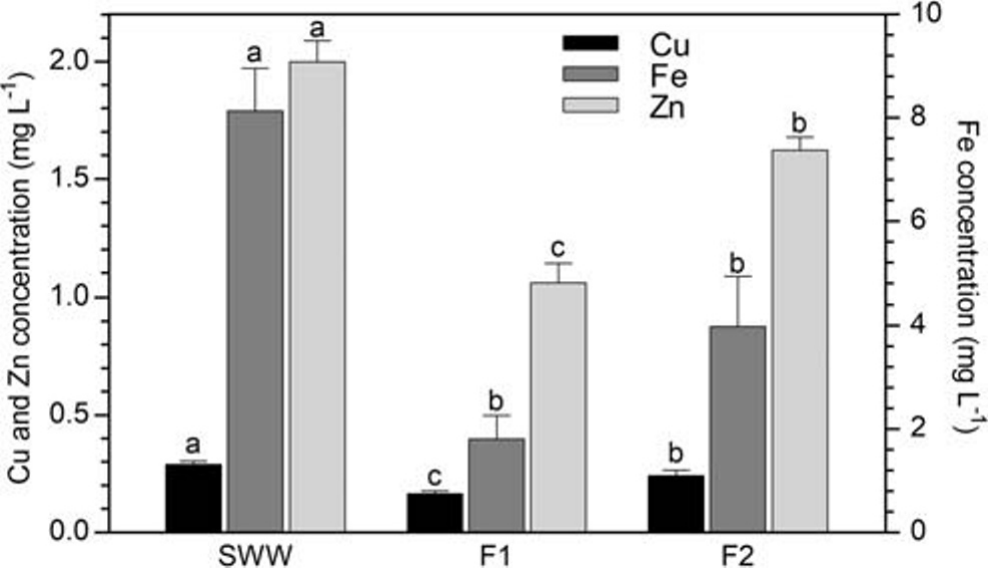
Metal concentration (mg L^−1^) in the synthetic water before (SWW) and after the percolation (F1 and F2) through the regenerated column system (mean of 15 replicates ± SE). Significant differences among the filtrations appear with different letters (at least *p* < 0.05)

The reusability of regenerated biosorbent was tested by performing new filtrations with SWW through the column system. As shown in Fig. 6, the regenerated biomass was still able to remove metal ions, since the residual concentrations in SWW after each filtrations (F1 and F2) were significantly decreased. In particular, for all the metals, the lowest values were found after the first filtration (F1), whereas after the second one (F2), the removal capacity was lower. Considering the Italian law, the concentrations determined in SWW after filtration were similar to the permitted emission limit values (Legislative Decree 152/06) only in the case of Cu and Fe after F1. In all the other cases, the values resulted above such threshold limits. However, such result would represent a small issue if the outlet of the column system is then destined to further treatments (e.g., through CW).

**Fig. 7:**
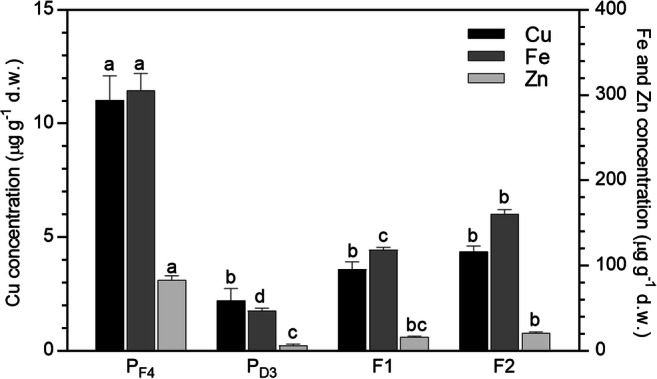
Metal concentration (μg g^−1^) in the regenerated *P. australis* biosorbent after two new percolations (F1 and F2) of SWW through the column system (mean of 15 replicates ± SE). The metal concentration in the *P. australis* biomass before (*P*_F4_) and after regeneration with HCl (*P*_D3_) is also reported in the graph. Significant differences among *P*_F4_, *P*_D3_, and the filtrations appear with different letters (at least *p* < 0.05)

Considering the metal concentrations in biosorbent after new filtrations (Fig. 7), starting from the regenerated biomass (*P*_D3_), in all cases, the values significantly increased both after F1 and after F2. Nonetheless, the concentrations obtained were always significantly lower than the ones reached in the first experiment after F1 and F2, and well far from the values reached after 4 consecutive filtrations (*P*_F4_). Such decrease in the sorption capacity after the regeneration is even more evident looking at the percentages of removal (Table 3), which were always significantly lower in respect to the ones found in the first use of the biosorbent. Therefore, despite the regenerated biosorbent proved to have still sorption capacity, the removal efficiency was decreased, probably due to possible biosorbent damage during desorption process, as already reported in literature for consecutive sorption-desorption cycles in column systems (Areco et al. 2012; Abdolali et al. 2017).

**Table 3:** Percentages of metal removal of *P. australis* regenerated biomass (mean of 15 replicates ± SE) after each filtration (F1 and F2) through the column system

Metals									
	Cu			Fe			Zn		
*R*%_SWW_									
F1	43	±	4 aB	74	±	5 aA	46	±	4 aB
F2	27	±	4 bAB	43	±	10 bA	17	±	3 bB
*R*%_biomass_									
F1	41	±	7 aB	75	±	3 aA	46	±	3 aB
F2	23	±	3 bB	44	±	3 bA	20	±	3 bB

Significant differences appear with different letters, small for the comparison among the filtrations and capital for the comparison among the metals (at least *p* < 0.05)

## Conclusions

A renewable biosorbent based on *Phragmites australis* dried biomass is here proposed to optimize the performance of a CW for the treatment of landfill leachates. Following the circular economy approach, the *P. australis* biomass was recycled from the annual mowing of the CW, combining the advantages of the improvement of wastewater remediation with an innovative way to manage and recycle the harvested plants, usually considered a waste.

The biomaterial showed an interesting sorption capacity for Cu, Fe, and Zn, both in batch and in column mode, especially for Fe ions. The immobilization of the biosorbent in column filters induced some improvement in the removal efficiency, and, in addition, this operation mode has the advantage of being much more suitable for practical applications than a batch process. Therefore, our sustainable column system filled with recycled *P. australis* biomass without any chemical modification showed a good potential in the metal removal from synthetic wastewater. Further studies are necessary to improve the reusability of the biosorbent and to optimize the technology using real wastewater.

## Data Availability

Data subject to third party restrictions.
